# Goal-directed haemodynamic therapy during elective total hip arthroplasty under regional anaesthesia

**DOI:** 10.1186/cc10246

**Published:** 2011-05-30

**Authors:** Maurizio Cecconi, Nicola Fasano, Nicola Langiano, Michele Divella, Maria G Costa, Andrew Rhodes, Giorgio Della Rocca

**Affiliations:** 1 Department of Anaesthesia and Intensive Care Medicine, Dipartimento di Scienze Mediche, Sperimentalie Cliniche, University of Udine, Piazzale Santa Maria della Misericordia, I-33100 Udine, Italy; 2 Department of General Intensive Care, St George's Hospital, Blackshaw Road, London SW17 0QT, UK

## Abstract

**Introduction:**

Total hip replacement is one of the most commonly performed major orthopaedic operations. Goal-directed therapy (GDT) using haemodynamic monitoring has previously demonstrated outcome benefits in high-risk surgical patients under general anaesthesia. GDT has never been formally assessed during regional anaesthesia.

**Methods:**

Patients undergoing total hip replacement while under regional anaesthesia were randomised to either the control group (CTRL) or the protocol group (GDT). Patients in the GDT group, in addition to standard monitoring, were connected to the FloTrac sensor/Vigileo monitor haemodynamic monitoring system, and a GDT protocol was used to maximise the stroke volume and target the oxygen delivery index to > 600 mL/minute/m^2^.

**Results:**

Patients randomised to the GDT group were given a greater volume of intravenous fluids during the intraoperative period (means ± standard deviation (SD): 6,032 ± 1,388 mL vs. 2,635 ± 346 mL; *P *< 0.0001), and more of the GDT patients received dobutamine (0 of 20 CTRL patients vs. 11 of 20 GDT patients; *P *< 0.0003). The GDT patients also received more blood transfused during the intraoperative period (means ± SD: 595 ± 316 mL vs. 0 ± 0 mL; *P *< 0.0001), although the CTRL group received greater volumes of blood replacement postoperatively (CTRL patients 658 ± 68 mL vs. GDT patients 198 ± 292 mL; *P *< 0.001). Overall blood consumption (intraoperatively and postoperatively) was not different between the two groups. There were an increased number of complications in the CTRL group (20 of 20 CTRL patients (100%) vs. 16 of 20 GDT patients (80%); *P *= 0.05). These outcomes were predominantly due to a difference in minor complications (20 of 20 CTRL patients (100%) vs. 15 of 20 GDT patients (75%); *P *= 0.047).

**Conclusions:**

GDT applied during regional anaesthesia in patients undergoing elective total hip replacement changes intraoperative fluid management and may improve patient outcomes by decreasing postoperative complications. Larger trials are required to confirm our findings.

**Trial registration:**

SRCTN11616985

## Introduction

Total hip replacement (THR) surgery is one of the most commonly performed major orthopaedic operations. Because of the increasing incidence of this type of surgery, a recent review article [1] considered it to be the operation of the century. When performed in elderly patients with comorbidities, it carries a significant burden of complications and mortality, with revision surgery having the highest risk (mortality up to 10%) [2-4]. Strategies that may reduce the burden of complications are therefore warranted.

The targeted administration of intravenous fluids, blood and vasoactive agents is commonly referred to as goal-directed therapy (GDT). GDT has been proven to reduce complications and mortality when performed in many scenarios [5-10] that include hemiarthroplasty for fractured neck of the femur [7,9]. The role of GDT in elective THR surgery and its possible impact on reducing complications and mortality have not been investigated to date. In recent years, the focus on postsurgical outcomes has been mostly on reducing mortality rather than on preventing complications. Recently, authors have demonstrated that long-term mortality following hospital discharge is associated with the development of a perioperative complication. It was our view, therefore, that strategies that decrease postoperative complications should be sought not just in high-risk patients but also in groups of patients who traditionally have been perceived to be at lower risk of mortality but have a significant risk of complications. We therefore decided to study patients undergoing elective THR surgery. In addition, THR surgery is commonly performed while the patient is under regional anaesthesia [11], which has some purported advantages, including a reduction in deep venous thrombosis and pulmonary embolism [12]. GDT has never been formally assessed while patients are under regional anaesthesia. This study therefore was undertaken to test the feasibility of applying GDT during THR surgery in non-high-risk surgical patients under regional anaesthesia to investigate whether GDT would lead to improved patient outcomes. We also wanted to uncover the implications of spinal blockade on intravenous fluid requirements and patients' haemodynamic status during GDT.

## Materials and methods

This study was approved by the Local Research Ethics Committee of Azienda Ospedaliero Universitaria. Adult patients scheduled for elective total hip arthroplasty under regional anaesthesia were screened for enrolment into the study by a member of the research team. Exclusion criteria included planned admission to a high-dependency intensive care setting in the postoperative period (all patients were scheduled to go back to the general orthopaedic ward from the surgical theatres), a contraindication to regional anaesthesia or the patient's refusal of consent.

### Protocol

This study was a randomised, controlled, single-centre trial conducted at the Azienda Ospedaliero Universitaria, Udine, Italy (ISRCTN11616985). Patients were screened for eligibility by a member of the research team who also obtained patients' written informed consent before surgery. Both the patients enrolled and the clinical teams caring for the patients were blinded to the study arm. Patients were randomised into one of two groups by using a concealed envelope technique: the standard haemodynamic therapy control group (CTRL group) or the GDT group. Patients remained in these two groups for the duration of surgery and for a further one hour in the postoperative recovery room. The protocol was finished at the end of this hour, and all patients then received standard postoperative care as directed by the clinical teams. All patients were then discharged from the recovery room back to the general orthopaedic ward according to standard practise in our hospital.

All patients were sedated with 0.02 mg/kg midazolam. Spinal anaesthesia was performed at the L3-L4 level with 15 mg of levobupivacaine at a 0.5% concentration. All patients underwent urinary catheterisation. Surgery commenced after spinal anaesthesia was deemed to be optimal. All patients received 10 mg of oxycodone two hours before surgery and every twelve hours following the operation for the next four days. Other analgesics given included regular paracetamol (1 g every 6 hours) for the first five days and tramadol and ketorolac as rescue analgesics when necessary.

All patients were monitored according to currently recommended standards during the intraoperative and immediate postoperative periods. In addition, for this study, all patients had their arterial blood pressure measured and recorded via an indwelling radial arterial catheter. Patients in both groups were kept normothermic (body temperature > 36.5°C), and the arterial oxygen saturation of haemoglobin (SaO_2_) was maintained above 94%. All patients received 10 mL/kg/hour of Ringer's lactate solution from 30 minutes prior to induction of anaesthesia through the end of surgery, when the rate was decreased to 100 mL/hour. In addition, boluses of 6% hydroxyethyl starch (HES) 130/0.4 (Voluven Fresenius Kabi, Bad Homburg, Germany) were given to the GDT group or the CTRL group according to the study arm to which the patient was allocated.

### Control group protocol

**Figure 1:**
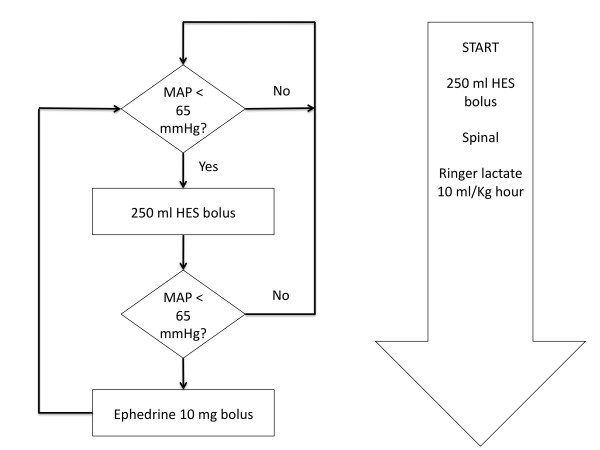
**Control group fluid management**. MAP = mean arterial pressure; HES = hydroxyethyl starch.

Patients randomised to the CTRL group received the standard therapy used at our institution prior to the study. Ringer's lactate solution at 10 mL/kg/hour was started 30 minutes prior to the induction of anaesthesia. An initial bolus of 250 mL of HES was given before the induction of anaesthesia. Boluses of intravenous colloid (250 mL) were administered if the patient's mean arterial pressure (MAP) fell below 65 mmHg. At the discretion of the attending anaesthetist, boluses of 10 mg of ephedrine were recommended for patients with severe hypotension that was not responding to fluid challenges (Figure 1).

### Protocol group

Patients randomised to the GDT group were connected to the FloTrac sensor/Vigileo haemodynamic monitoring system (Edwards Lifesciences, Irvine, CA, USA). The FloTrac sensor is the specific transducer that is required to connect the arterial line to the Vigileo monitor. After inputting demographic data (patient age, weight and sex), we used the pulse pressure algorithm (version 1.07) to track continuously stroke volume (SV) and cardiac output (CO). The oxygen delivery index (DO_2_I) was then calculated by inputting the haemoglobin concentration and SaO_2 _into standard equations.

**Figure 2:**
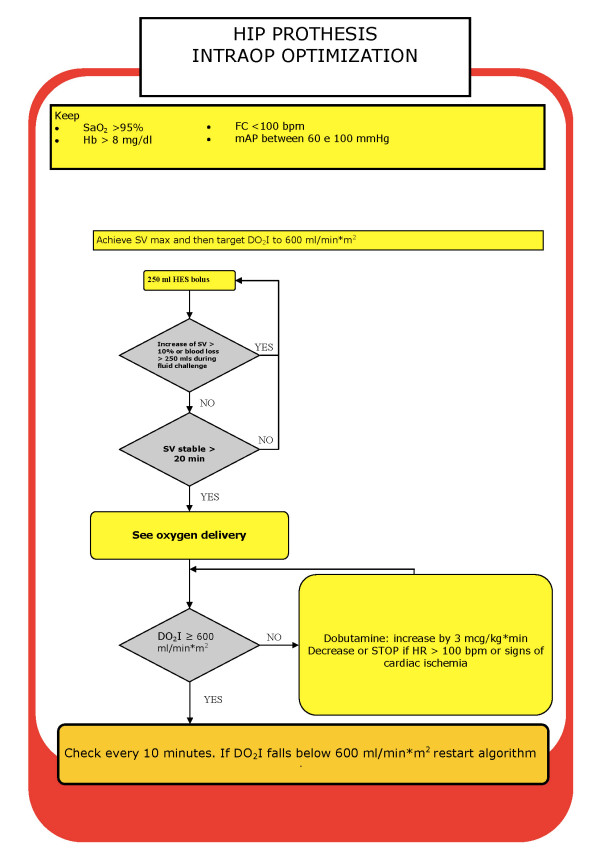
**Intraoperative goal-directed therapy protocol (GDT)**. GDT = goal-directed therapy group; SV max = maximum stroke volume; DO_2_I = oxygen delivery index; HES = hydroxyethyl starch; HR = heart rate; bpm = beats/minute.

The protocol for the haemodynamic management of the GDT patients was based on the postoperative GDT protocol of St George's Hospital [13]. In the present study, SV was first maximised with fluid challenges. Boluses of 250 mL of HES were administered until the SV failed to increase by a factor of 10%. If 25 mL/kg HES had been given before SV maximisation was achieved, fluid challenges were then performed with 250 mL boluses of Ringer's lactate solution. If at this stage the DO_2_I was not greater than 600 mL/m^2^, then dobutamine was started at a dose of 3 μg/kg/minute and increased by the same increment every 20 minutes to reach the described target. To ensure that dobutamine was started only under optimal conditions, blood samples were taken every 30 minutes and blood transfusions were used to maintain a haemoglobin concentration over 10 g/dL (Figure 2).

### Data collection and follow-up

All patients were scored for their American Society of Anesthesiologists (ASA) classification [14] prior to surgery. All patients were followed up until hospital discharge. All complications including deaths were recorded. These were defined *a priori *and were diagnosed by the clinical teams who were blind to study arm allocation and were then confirmed by the research team, We defined and classified postoperative complications according to a modified version of the Postoperative Morbidity Survey used by Bennett-Guerrero *et al*. [15]. The definitions of complications are described in Additional file 1[15,16].

### Statistical analysis

The incidence of postoperative complications in this elective population is not well known. To power the study, we looked at differences in fluid management. This study was therefore powered to demonstrate a difference in total intravenous fluids administered between the two study arms. Historical data at this institution demonstrated that in a similar cohort of patients, the mean (± SD) total fluid administered during the operative period and for one hour in the postoperative phase was 2,600 ± 600 mL. We estimated that a 500-mL difference in total fluid input would be relevant to the patients, and therefore 38 patients (19 in each group) would need to be enrolled to detect this difference with 80% power at a *P *value < 0.05. To allow for a 5% error in these calculations, we set out to enrol 20 patients into each study arm.

Data were analysed using GraphPad Prism 4.0 software (GraphPad Software, Inc., La Jolla, CA, USA). Data are presented as means (± SD) when normally distributed and as medians (interquartile range (IQR)) when not normally distributed. Continuous data were compared by using either the Mann-Whitney *U *test or the Wilcoxon test as appropriate. Categorical data were compared using Fisher's exact test. A *P *value < 0.05 was considered statistically significant.

## Results

Forty patients were enrolled into the study between March 2008 and March 2009. All screened patients were randomised, and complete follow-up was achieved for all randomised patients. All patients underwent spinal anaesthesia without any complications. All patients went back to the general ward after the end of the operation. All patients completed the study and are included in the intention-to-treat analysis. A consort chart of the flow of patients is presented in Additional file 2. The demographic characteristics and baseline haemodynamics of the patients are summarised in Table 1. The two groups were similar in terms of all demographic data. In particular, the patients were well matched for severity of illness and background comorbidities as assessed by their ASA scores.

**Table 1:** Demographic data and surgical characteristics of the patients at baseline

Demographics	Control	GDT
Number of patients	20	20
Median age (IQR), years	63 (58 to 72)	69 (61 to 76)
Male sex, *n *(%)	9 (45%)	14 (70%)
Mean weight (± SD), kg	81 ± 16	80 ± 13
Median height (± SD), cm	170 (167 to 173)	170 (166 to 180)
Median ASA grade (IQR)	2 (2 to 2)	2 (2 to 2)

ASA: American Society of Anesthesiologists; GDT: goal-directed therapy.

### Haemodynamic management

Patients randomised to the GDT group received a greater volume of fluids during the intraoperative period (mean (± SD) 6,032 ± 1,388 mL vs. 2,635 ± 346 mL; *P *< 0.0001) (Table 2). In particular, the GDT group was given more crystalloid (mean (± SD) 3,358 ± 825 mL vs. 2,100 ± 361 mL; *P *< 0.0001), more colloid (mean (± SD) 2,079 ± 858 mL vs. 535 ± 86 mL; *P *< 0.0001) and more blood (mean (± SD) 595 ± 316 mL vs. 0 ± 0 mL; *P *< 0.0001). The GDT patients had a higher urine output (median (IQR) 1225 (650 to 1375) mL vs. 300 (100 to 475) mL; *P *< 0.0001) during this period and ended up with a more positive fluid balance (3,565 ± 879 mL vs. 1403 ± 401 mL; *P *< 0.0001). More patients in the GDT group than in the CTRL group received dobutamine (11 patients (55%) vs. 0 patients (0%); *P *< 0.0003) (Table 2).

**Table 2:** Intraoperative fluid management as well as perioperative blood consumption intraoperatively, postoperatively and overall

Fluid management and blood transfusion	CTRL group	GDT group	*P *value
Intraoperative period			
Dobutamine, *n *(%)	0 (0%)	11 (55%)	0.0003
Dobutamine dose, μg/kg/minute	n/a	4 ± 1	n.a.
Mean Crystalloids (± SD), mL	2,100 ± 361	3,358 ± 825	< 0.0001
Mean Colloids (± SD), mL	535 ± 86	2,079 ± 858	< 0.0001
Mean Blood (± SD), mL	0	595 ± 316	0.0003
Mean Total input (± SD), mL	2,635 ± 346	6,032 ± 1,388	< 0.0001
Median Urine output (IQR), mL	300 (100 to 475)	1225 (650 to 1375)	< 0.0001
Median Blood loss (IQR), mL	900 (800 to 975)	900 (750 to 1200)	n.s.
Mean Fluid balance (± SD), mL	1,403 ± 401	3565 ± 879	< 0.0001
Perioperative blood transfusion requirements, mL			
Mean Intraoperative blood transfusion (± SD)	0	565 ± 332	0.0003
Mean Postoperative blood transfusion (± SD)	658 ± 302	198 ± 292	0.0004
Mean Total blood transfusion (± SD)	658 ± 302	792 ± 480	n/s

CTRL: control; GDT: goal-directed therapy; n/a: not applicable; n/s: not significant.

*P *values are reported if statistically significant.

**Figure 3:**
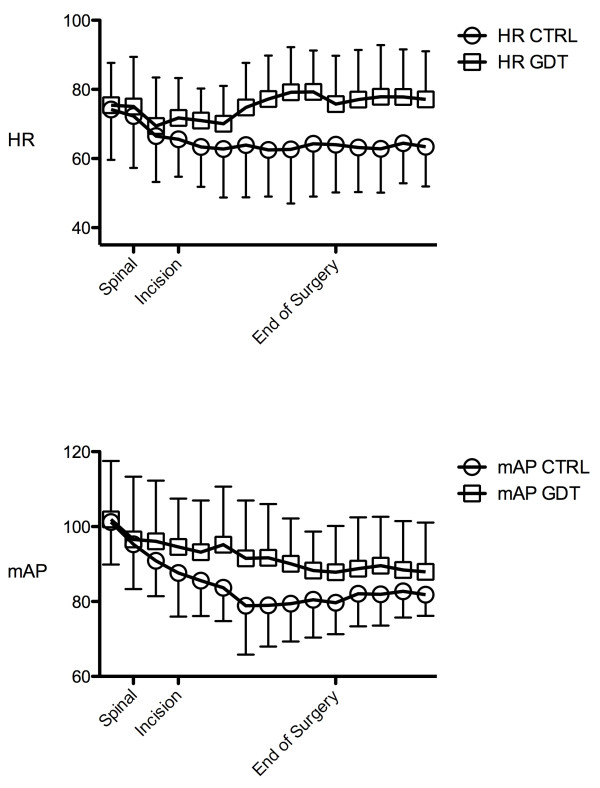
**Trends in heart rate and mean arterial pressure between control group and goal-directed therapy group**. HR = heart rate; mAP = mean arterial pressure; GDT = goal-directed therapy group; CTRL = control group.

The patients' baseline haemodynamics prior to induction of spinal anaesthesia were similar between the two groups. No patient required a vasoconstrictor to maintain MAP, which was maintained above 65 mmHg at all time points in both groups. On the basis of repeated measures analysis of variance, the GDT patients had a higher mean heart rate and a higher MAP (Figure 3) than CTRL patients over the course of the operations. By the end of the operations, both the mean heart rate (78 ± 15 beats/minute vs. 63 ± 12 beats/minute; *P *= 0.002) and the MAP (89 ± 13 mmHg vs. 82 ± 6 mmHg; *P *= 0.03) were higher in the GDT group.

At baseline in the GDT group, five patients already had a DO_2_I > 600 mL/minute/m^2^. During the study period, 13 (65%) of 20 patients reached a DO_2_I > 600 mL/minute/m^2 ^in the GDT group. All but one of seven patients who did not reach the DO_2_I target were started on dobutamine therapy. The one patient was not started on dobutamine therapy because he reached SV maximisation only at the end of the study period. There were no reported complications directly related to GDT.

### Outcomes

No patients died within the 28 days of enrolment in the study. There were no differences in time to hospital discharge between the CTRL group and the GDT group (median 10 days (IQR 9 to 11 days) vs. median 10 days (IQR 9 or 10 days); *P *= 0.618).

A reduced number of patients in the GDT group compared to the CTRL group developed postoperative complications (16 patients (80%) vs. 20 patients (100%), respectively; *P *= 0.053). The median number of complications per patient was lower in the GDT group than in the CTRL group (median 4 complications/patient (IQR 3 to 4) vs. median 1 complication/patient (IQR 0 to 2 complications), respectively; *P *< 0.0001). These complications were subdivided into minor and major complications according to predefined criteria (Tables 3 and 4).

**Table 3:** Minor complications during hospital stay in the control group and the goal-directed therapy group

Minor complications	CTRL, *n*	GDT, *n*
Uncomplicated infections		
Abdominal	1	0
Urinary	5	3
Cardiovascular		
Hypotension	44	13
Anaemia^a^	18	7
Total	68	23

CTRL: control; GDT; goal-directed therapy.

^a^anaemia requiring blood transfusion.

**Table 4:** Major complications during hospital stay in the control group and the goal-directed therapy group

Major complications	CTRL, *n*	GDT, *n*
Infection		
Pneumonia	0	0
Respiratory		
Pulmonary embolism	1	1
Cardiovascular		
Tachyarrhythmias^a^	4	0
Acute coronary syndrome	2	0
Renal		
Oliguria, acute renal failure	0	0
Total	8	1

CTRL: control; GDT: goal-directed therapy.

^a^arrhythmias requiring pharmacological interventions.

Significantly fewer patients in the GDT group than in the CTRL group developed minor complications (15 (75%) of 20 patients vs. 20 (100%) of 20 patients, respectively; relative risk (RR) 1.3 (1.04 to 1.72) and *P *= 0.047). Significant postoperative hypotension was especially common in the CTRL group, with 19 (95%) of 20 patients having at least one episode of systolic blood pressure < 90 mmHg that required urgent fluid administration (500 mL of HES) following discharge to the general orthopaedic ward. This was a much higher proportion than that in the GDT group (9 (45%) of 20 patients; RR 2.1 (1.3 to 3.5) and *P *< 0.0001). The median number of minor complications per patient was also lower in the GDT group than in the CTRL group (1/patient (IQR 0 to 2) vs. 4/patient (IQR 3 to 4), respectively; *P *< 0.0001).

Five patients (25%) in the CTRL group had major complications vs. one patient (5%) in the GDT group, although this difference did not reach statistical significance (*p = *0.076) (Table 4). No patient (0%) in the GDT group had a major cardiac complication, while five patients (25%) in the CTRL group did (*P *= 0.047).

There was a trend towards a reduction in postoperative nausea and vomiting (PONV) in the GDT group compared to the CTRL group (9 patients (45%) vs. 4 patients (20%); *P *= 0.176), although this finding did not reach statistical significance. Among the total number of PONV episodes, there was a reduction in the episodes of nausea per patient in the GDT group (0.7 ± 0.2 vs. 0.15 ± 0.1; *P *= 0.02; data expressed as mean (± SD)).

## Discussion

This study demonstrates that GDT is feasible during regional anaesthesia in awake patients. The protocol that we used was associated with a significantly greater use of intraoperative fluids and inotropic agents, with a consequent reduction in postoperative minor complications and major cardiovascular complications. We chose an approach similar to that used by Pearse *et al*. [13] in their postoperative GDT study, in which a DO_2_I of 600 mL/m^2 ^was targeted after SV maximisation. Our protocol group was monitored with the Vigileo monitor. This allowed us to give fluid in response to changes in SV measured in real time. This is the first study in which a SV maximisation approach was used during spinal anaesthesia in awake patients.

It is important to note the great differences in fluid administration between the GDT and CTRL groups in this study. Our study was powered to reflect 500-mL differences in fluid balance, while we found that the GDT group received more than twice the fluid input of the CTRL group. We chose to direct our CTRL group according to local standard practise. Our CTRL patients received a total average fluid input of 2.6 L during the protocol period. This group, therefore, were not subject to a 'restricted' approach to fluid management; in fact, they received a considerable volume of fluid. There are few specific data available for comparison regarding elective hip arthroplasty patients. Holte *et al*. [17] studied the differences in outcomes between liberal and restrictive strategies during elective knee arthroplasty. Patients in the restrictive group in their study received, on average, 1.7 L of fluid in comparison to 4.2 L in the protocol arm. Importantly, we did not want to look at a restrictive versus liberal fluid infusion strategy. In our study, we aimed at looking at differences in individualised haemodynamic management between two protocols.

An explanation to the high fluid intake may be that spinal anaesthesia is responsible for relative hypovolaemia by abolishing the sympathetic tone, therefore creating increased venous capacitance. The biggest response to fluid challenges in terms of increases in cardiac index occurred just before the induction of spinal anaesthesia and the biggest decreases occurred immediately afterwards. The effect of spinal anaesthesia on cardiac index has not been well described in the literature. Dyer *et al*. [18] found that spinal anaesthesia was linked to a drop in blood pressure with minimal impact on flow. Clearly, if our results are confirmed in future studies, then the haemodynamic impact of spinal anaesthesia is bigger than has traditionally been expected.

GDT has been shown to be effective when applied in high-risk surgical patients during the perioperative period. It is often referred to as early GDT when it is started immediately after surgery [13] or immediately after the recognition of shock [11]. The timing of the intervention is especially important. When started late, after the establishment of organ failure, GDT does not improve patient outcomes [19,20]. In the context of high-risk surgical patients, many studies have assessed the use of GDT intraoperatively [7-9] and demonstrated a benefit for the patient [21,22].

To our knowledge, few studies have directly set out to assess the effects of GDT in low-risk groups of patients. A recent meta-analysis demonstrated, however, that GDT is still beneficial in terms of reducing both mortality and morbidity when used in lower-risk groups [22]. Previous authors have aimed to achieve SV maximisation alone in lower-risk groups, especially during colorectal surgery [8,23] and following surgery for fractured neck of the femur [7,9]. Pearse *et al*. [4] defined 'high-risk surgical patients' as those patients who have an expected mortality higher than 5%. GDT is traditionally applied to this subgroup of patients. Despite this, few studies have demonstrated a reduction in mortality. This benefit was initially demonstrated by Shoemaker *et al*. [5] and Boyd *et al*. [6], while more recent studies have shown reductions in postoperative complications and length of hospital stay [13]. Postoperative complications may have a big impact on long-term outcomes and affect long-term mortality even after hospital discharge [24].

We found a large reduction in minor postoperative complications and major cardiovascular complications. We cannot exclude that our results may just be a reflection of monitoring variables (SV, CO and DO_2_I) that normally are not monitored in this population. It is important to remember, though, that there are several examples in the literature where the simple use of CO monitors (the pulmonary artery catheter, for instance) without a protocol has not provided any benefit to the patients [25,26]. On the other hand, a benefit has been observed when a CO monitor has been used as part of a preemptive strategy to optimise the haemodynamics in surgical patients. This has recently been demonstrated in a meta-analysis by Hamilton *et al*. [22]. We think that our results are explained more by CO monitoring and GDT than by CO monitoring alone.

Despite a reduction in total number of complications, we did not find any reduction in hospital length of stay or mortality. It could be argued that the lack of differences in length of stay implies that the complications were neither serious nor relevant. The reason behind this possibility, however, relates to the fact that all patients in this study at our institution had a planned length of stay of 10 days, which was not shortened even in the absence of need. This limit on length of hospital stay has been described before in the literature [16,27,28].

Clearly this is important, but to demonstrate differences in either length of stay or mortality, a much larger group of patients must be studied to provide adequate power. Another important finding of this study has to do with perioperative blood utilisation. The GDT group received most of the perioperative blood during the operation, while the CTRL group received more blood postoperatively. Interestingly, the overall blood utilisation was the same in both groups.

## Conclusions

Intraoperatively, GDT during THR surgery in which spinal anaesthesia is used in awake patients is feasible and leads to a different type of haemodynamic management. Our results suggest that GDT may improve outcomes in this population by decreasing the rate of minor postoperative complications. Larger trials are required to confirm our findings.

## Key messages

• GDT during spinal anaesthesia and awake patient is feasible using an arterial line and the Vigileo monitor.

• Intraoperative GDT during THR surgery decreases postoperative complications.

## Abbreviations

CTRL: control; DO_2_I: oxygen delivery index; GDT: goal-directed therapy; HES: hydroxyethyl starch; IQR: interquartile range; PONV: postoperative nausea and vomiting; SV: stroke volume; THR: total hip replacement.

## Competing interests

MC and AR received lecturing fees from Edwards Lifesciences, LiDCO and Cheetah Medical, as well as research support from LiDCO. The other authors do not have any conflicts of interest to disclose.

## Authors' contributions

MC planned the study, collected data and analysed and wrote the manuscript. NF planned the study and collected data. NL collected the data. MG and MD collected data, supervised the study and wrote the manuscript. AR supervised the statistical analysis and wrote the manuscript. GDR planned the study, supervised the data collection and statistical analysis and wrote the manuscript.

## Supplementary Material

Additional file 1 **Modified Postoperative Morbidity Survey**. Classification of the complications recorded according to the modified Postoperative Morbidity Survey (POMS). Original POMS data have been modified to separate major and minor complications. Click here for file

Additional file 2 **Consort diagram of the study**. Consort diagram of patients enrolled into the study. GDT = goal-directed therapy group; CTRL = control group. Click here for file
