# Supplementary material for: Goal-directed haemodynamic therapy during elective total hip arthroplasty under regional anaesthesia
Source: Crit Care. 2011 May 30;15(3):R132. doi: 10.1186/cc10246 (PMC3218998; doi:10.1186/cc10246)
Supplement: Additional file 1 — Modified Postoperative Morbidity Survey. Classification of the complications recorded according to the modified Postoperative Morbidity Survey (POMS). Original POMS data have been modified to separate major and minor complications. [file cc10246-S1.DOC]

**Appendix 1 Significant Complications. Modifications of POMS used by Bennett-Guerrero[1].**

Complications were classified using the system described by Bennett-Guerrero and separated into either major or minor complications. They were defined as:

**Major Complications:**

- Infection (proven Infection, i.e. respiratory, urinary, abdominal, wound with signs of SEPSIS)
- Major cardiovascular complications (acute coronary syndrome, pulmonary oedema, arrhythmia),
- Pulmonary Embolism,
- Renal Failure,
- Anaemia requiring blood transfusion in the presence of shock.

**Minor Complications:**

- Hypotension (systolic blood pressure < 90 mmHg) requiring fluid boluses,
- Uncomplicated infections (not requiring intra-venous antibiotic therapy and with no signs of sepsis, i.e. uncomplicated urinary tract infections),
- Anaemia requiring blood transfusions in the absence of shock.

**PONV:**

Postoperative Nausea and Vomiting, originally inserted among complications in the Bennett Guerrero classification were considered by us as a different category (nor major neither minor complications, but simply PONV)
