# Supplementary figures and images for: Goal-directed haemodynamic therapy during elective total hip arthroplasty under regional anaesthesia
Source: Crit Care. 2011 May 30;15(3):R132. doi: 10.1186/cc10246 (PMC3218998; doi:10.1186/cc10246)

Appendix 2. Consort Diagram of Study.


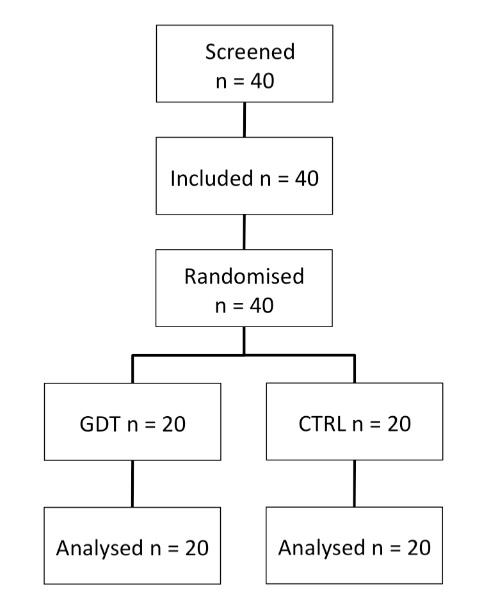

Supplement: Additional file 2 — Consort diagram of the study. Consort diagram of patients enrolled into the study. GDT = goal-directed therapy group; CTRL = control group. [file cc10246-S2.DOC]
